# A pilot evaluation of the diagnostic accuracy of ChatGPT-3.5 for multiple sclerosis from case reports

**DOI:** 10.1515/tnsci-2022-0361

**Published:** 2024-12-24

**Authors:** Anika Joseph, Kevin Joseph, Angelyn Joseph

**Affiliations:** Health Sciences Program, University of Ottawa, 75 Laurier Ave E, Ottawa, ON K1N 6N5, Canada; Biomedical Science Program, University of Ottawa, 75 Laurier Ave E, Ottawa, ON K1N 6N5, Canada; Merivale High School, 1755 Merivale Rd, Nepean, ON K2G 1E2, Canada

**Keywords:** artificial intelligence, multiple sclerosis, case reports, legal

## Abstract

The limitation of artificial intelligence (AI) large language models to diagnose diseases from the perspective of patient safety remains underexplored and potential challenges, such as diagnostic errors and legal challenges, need to be addressed. To demonstrate the limitations of AI, we used ChatGPT-3.5 developed by OpenAI, as a tool for medical diagnosis using text-based case reports of multiple sclerosis (MS), which was selected as a prototypic disease. We analyzed 98 peer-reviewed case reports selected based on free-full text availability and published within the past decade (2014–2024), excluding any mention of an MS diagnosis to avoid bias. ChatGPT-3.5 was used to interpret clinical presentations and laboratory data from these reports. The model correctly diagnosed MS in 77 cases, achieving an accuracy rate of 78.6%. However, the remaining 21 cases were misdiagnosed, highlighting the model’s limitations. Factors contributing to the errors include variability in data presentation and the inherent complexity of MS diagnosis, which requires imaging modalities in addition to clinical presentations and laboratory data. While these findings suggest that AI can support disease diagnosis and healthcare providers in decision-making, inadequate training with large datasets may lead to significant inaccuracies. Integrating AI into clinical practice necessitates rigorous validation and robust regulatory frameworks to ensure responsible use.

## 1 Introduction

The integration of artificial intelligence (AI) in diagnostics has shown promise in various fields such as radiology, pathology, and ophthalmology [1]. Within the realm of disorders, AI has been utilized to diagnose Alzheimer’s and Parkinson’s diseases, and brain tumors [2]. In the field of medical diagnostics, AI applications can be generally classified into two categories – medical imaging AI and generative AI. Medical imaging AI has garnered substantial attention with a focus on analysis of visual data from magnetic resonance imaging (MRI), X-rays, and computed tomography to detect abnormalities and disease-related patterns. In contrast, generative AI such as chat generative pre-trained transformer (ChatGPT)-3.5, relies on large language models (LLMs) trained on extensive datasets [3]. While medical imaging AI excels at visual pattern recognition, generative AI brings a unique capability to interpret textual information. Despite the increasing use of LLMs in healthcare, reliance on AI tools to diagnose a condition may lead to medical negligence and liability [4]. This is validated by findings in the literature that the accuracy of AI tools such as ChatGPT, an LLM developed by OpenAI, for making medical diagnosis is only 57% [5].

The risk of misdiagnosis due to AI shortcomings is not merely theoretical. A 2021 study discovered that an AI system trained to identify COVID-19 from chest X-rays was detecting text markers on the images instead of analyzing the medical content. This shortcut caused the AI system to incorrectly classify many non-COVID X-rays as positive for the disease, emphasizing how AI can fail in critical diagnostic tasks [6].

An argument can be made for the use of AI because of its ability to predict the risk of breast cancer [7] and perhaps other diseases [8]. However, not using AI for the fear of relatively poor accuracy can also lead to physicians being accused of delivery of substandard care [9]. Therefore, there is a potential for medical negligence and liability to arise, which can have an impact on the ability of physicians to adopt AI into their practice and that of patients in fully trusting physicians to heal their suffering. In view of this paralytic state that both physicians and patients can face, there is a need for increased training datasets with patient clinical and laboratory data, particularly for diseases that are racially- ethnically- and gender-restricted.

The aim of this study was to determine the extent to which ChatGPT-3.5 misinterpreted patient data obtained at clinical presentation, laboratory, and histology with a diagnosis of multiple sclerosis (MS), which was chosen as a prototypic example in this study from many disorders whose diagnoses are amenable to AI.

Current studies regarding the diagnosis of MS through identification of lesions and prediction of disease progression have mainly centered on enhancing imaging techniques and utilizing biomarkers for early identification [10]; these processes are being automated with AI [11]. Eitel et al. showcased AI-powered deep learning algorithms that could accurately spot MS lesions in MRI scans, potentially simplifying the process and reducing the necessity for image analysis expertise [12]. Past studies on AI applications in diagnosing conditions have primarily focused on image interpretation and identifying patterns. For example, Yoo et al. employed networks to precisely classify MRI scans of MS patients [11].

The diagnosis of MS presents significant challenges for physicians due to the disease’s complex and variable nature. Traditional diagnostic methods, relying heavily on clinical evaluations and MRI findings, often lack the specificity needed to distinguish MS from other central nervous system inflammatory diseases [13]. Furthermore, a recent analysis from two clinics found that 18% of patients initially diagnosed with MS were later determined to have been misdiagnosed [14]. This gap necessitates the exploration of advanced diagnostic tools to improve the accuracy and efficiency of MS diagnosis, and AI offers transformative potential in this regard. While recent research has made strides in using deep learning techniques to analyze MRI scans and predict disease progression in various neurological conditions, the specific application of AI tools such as ChatGPT-3.5 to diagnose MS remains underexplored. Also, there is limited attention to the potential of LLMs in diagnosing MS from case reports that include clinical presentation and laboratory data [15].

The decision to focus on MS in this study was motivated by its diagnostic complexity, the significant rate of misdiagnosis, and the potential for AI to address these challenges. MS serves as a representative condition for exploring the utility of AI in healthcare because its diagnosis relies on synthesizing diverse data types, including imaging, clinical symptoms, and laboratory results.

Case histories are published in peer-reviewed journals and patient data are described qualitatively and quantitatively in a structured format that is amenable to analysis using LLM applications. Among several such applications, ChatGPT-3.5 stands apart from the others due to its convenience of utility, accessibility, and simplicity [16]. ChatGPT-3.5 provides a unique opportunity to explore the application of LLMs in MS diagnosis from case reports. Unlike image-based models, ChatGPT-3.5 analyzes and generates responses based solely on text [17] offering a distinct approach to understanding its diagnostic capabilities [18].

This study aimed to provide insights into the potential and limitations of LLMs in medical diagnostics by assessing ChatGPT-3.5’s performance in diagnosing MS using publicly available clinical presentations and laboratory data from published case reports, offering a more thorough evaluation of its efficacy and potential clinical applications. By demonstrating the accuracy of ChatGPT-3.5 for the clinical diagnosis of MS, we aimed to show that misinterpretations arising from the use of LLMs may affect patient safety. The findings will help determine the effectiveness of AI tools in interpreting textual clinical information, highlight potential risks, and guide future improvements in AI diagnostic practices by identifying areas of improvement for AI tools.

## 2 Methods

### 2.1 Study design

This study employed a retrospective analysis of 98 case reports published within the past 10 years. We utilized an LLM AI model to analyze these reports and generate diagnoses.

We selected a sample size of 98 reports based on precedent from prior ChatGPT studies, which utilized similar sample sizes to assess diagnostic capabilities. For example, one study analyzed 90 dermatology cases [19], while another evaluated 100 neuroradiology cases [20]. This sample size was chosen to align with existing methodologies, providing a balance between feasibility and the generation of meaningful insights.

Only true positive cases were presented to ChatGPT-3.5 as sensitivity was prioritized over the detection of false positives. In clinical practice, initial diagnostic tools that aid the practitioner are proceeded by more specific tests and procedures to confirm the initial diagnosis. Hence, the measurement of false positives is less critical as their impact is mitigated with additional testing to confirm the initial diagnosis from the tool.

### 2.2 Data collection

A search for case reports was conducted on PubMed [21]. The criteria for selection of case reports included two key factors: accessibility and timeframe. First, only free full-text case reports of MS were considered to ensure complete data availability for analysis by the LLM AI model. Second, only case reports published within the past 10 years (2014–2024) were considered to ensure data were current and reflective of the latest clinical practices and diagnostic criteria.

Both clinical presentations and laboratory findings were provided to the AI model. Clinical presentations included details on patient histories, symptoms, and disease progression. Laboratory findings included analysis of blood and cerebrospinal fluid for myelin basic protein, and immunoglobulin G.

**Figure 1:**
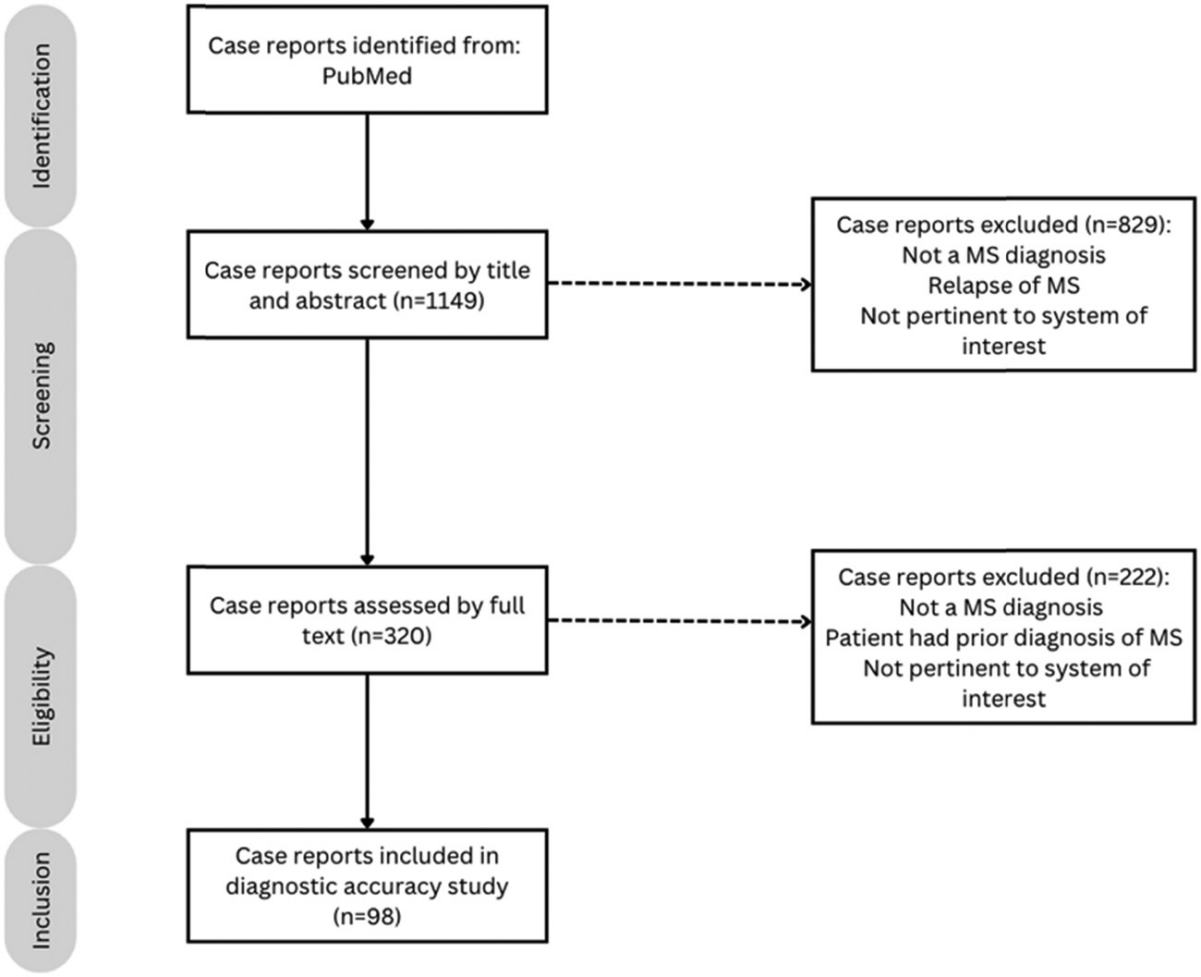
PRISMA flow chart detailing the method of inclusion and exclusion.

Case reports with a prior MS diagnosis were excluded to avoid biasing the AI model. A total of 98 cases were selected for this study (Figure 1).

### 2.3 AI model used

ChatGPT-3.5 was used as the AI diagnostic tool for this study. The model was prompted with clinical information and laboratory data from each case report. In instances of case reports with an explicit mention of an MS diagnosis, any reference to the diagnosis was removed to maintain the integrity of the study. In addition, any figures were excluded as ChatGPT-3.5 cannot process or interpret images.

The prompt used was “Disregarding the figure mentions - Diagnose this patient with one specific disease.”

ChatGPT-3.5’s memory function, which enables the model to remember details from previous interactions and provide more tailored answers, was disabled. This practice ensured that each diagnostic assessment was based solely on the clinical information in each individual case report, eliminating potential bias from prior interactions or cases.

Responses from ChatGPT-3.5 were represented as accurate diagnoses by all three investigators when the generated text clearly indicated that MS was the leading diagnosis for the presented case.

At the time of experimentation, ChatGPT-3.5 was employed, being the only available tool. However, ChatGPT-3.5 retired in 2024 and ChatGPT-4o, which is capable of analyzing text, images, and audio with enhanced diagnostic accuracy became available in the same year but was considered out of scope in the current study design and therefore not pursued.

### 2.4 Statistical analysis

A spreadsheet was used to systematically track each case report and record the diagnoses provided by the AI model, which were categorized as accurate or inaccurate. The accuracy of the AI model was determined by dividing the number of correct MS diagnoses by the total number of cases.

The model’s performance in terms of sensitivity, specificity, and accuracy was also analyzed. Sensitivity quantifies the proportion of true positives correctly identified, and therefore the proportion of cases where MS was correctly diagnosed by the AI model. Conversely, specificity measures the proportion of true negatives correctly identified. Specificity was calculated but since case reports without MS were not included in the dataset, specificity was deemed to be inapplicable in this study. Accuracy provides a comprehensive assessment of the AI model’s ability to correctly identify both positive and negative cases across the entire dataset.

## 3 Results

**Figure 2:**
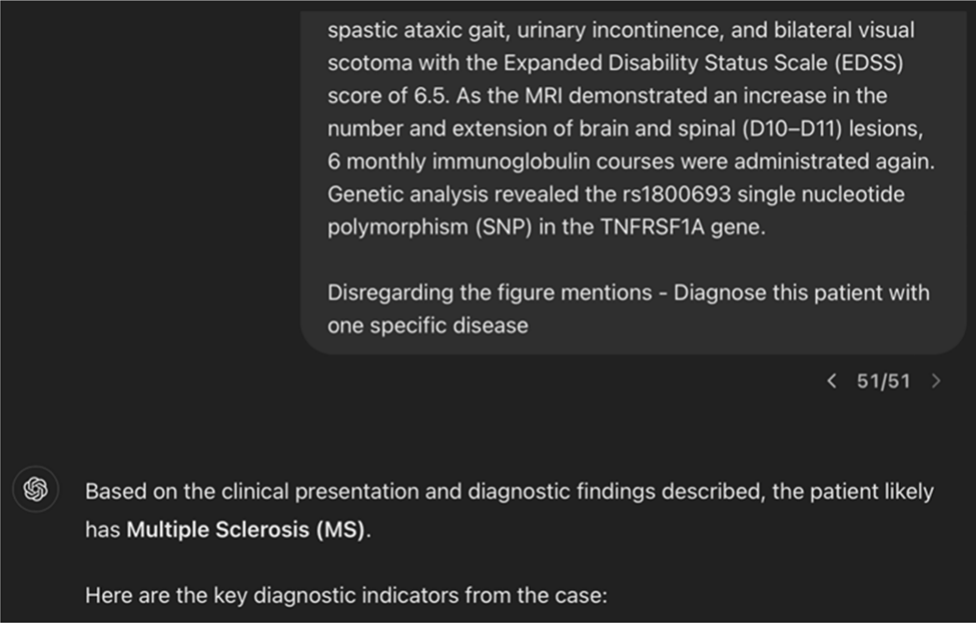
Screenshot of accurate diagnosis of MS by ChatGPT-3.5 through text generation based on case presentation.

**Figure 3:**
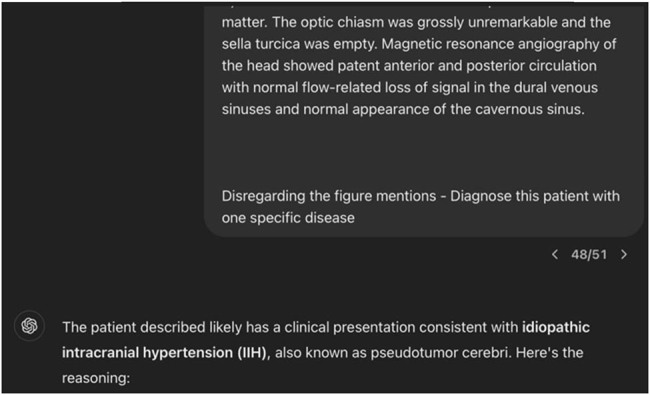
Screenshot of inaccurate diagnosis by ChatGPT-3.5 through text generation based on case presentation.

Our analysis showed that ChatGPT-3.5 correctly diagnosed MS in 77 out of 98 cases, yielding an accuracy of 78.6%. The sensitivity and specificity rates are shown in Table 1. Examples of accurate (Figure 2) and inaccurate (Figure 3) diagnoses of MS by ChatGPT-3.5 through text generation based on case presentation are shown.

**Table 1:** Sensitivity, specificity, and accuracy of diagnosis

Outcome of the diagnostic test	MS		
	As determined by the Standard of Truth		
	Positive	Negative	Row total
Positive	77	0	77
Negative	21	0	21
Column total	98	0	98

\[\text{Sensitivity}=\text{TP}/(\text{TP}+\text{FN})=0.77]\]


\[\text{Specificity}=\text{TN}/(\text{TN}+\text{FP})=\text{n/a}]\]


\[\text{Accuracy}=(\text{TN}+\text{TP})/(\text{TN}\hspace{.5em}+\hspace{.5em}\text{TP}+\text{FN}\hspace{.5em}+\hspace{.5em}\text{FP})=0.77]\]

Of the 21 inaccurate responses, the most common misdiagnoses were acute disseminated encephalomyelitis (*n* = 4) and neuromyelitis optica spectrum disorder (*n* = 3). Both these conditions can present with neurological symptoms similar to MS and therefore may contribute to diagnostic challenges for ChatGPT-3.5. The remaining 14 cases were misdiagnosed as various inflammatory, infectious, autoimmune, and rare disorders. However, all of these conditions can present with neurological symptoms that closely resemble those of MS, contributing to diagnostic inaccuracies.

## 4 Discussion

The purpose of this study was to evaluate the potential of ChatGPT-3.5 to diagnose MS when presented with text-only case reports. This study reveals the potential, and limitations of ChatGPT-3.5 for use in diagnosing MS when provided a dataset of case reports. The 98 case reports examined in this study were obtained from peer-reviewed journals published between 2014 and 2024, offering a diverse and recent dataset. These reports encompassed a wide range of MS presentations, including relapsing-remitting, primary progressive, and secondary progressive forms of the disease. The complexity of the cases varied from straightforward presentations to more challenging atypical presentations, allowing for a thorough assessment of ChatGPT-3.5’s diagnostic abilities.

Our results demonstrate the potential of ChatGPT-3.5 in diagnosing MS based on clinical presentations and laboratory data described in case reports. These results align with the growing body of evidence suggesting that AI, and specifically LLM models such as ChatGPT-3.5, can effectively interpret clinical and laboratory data to make accurate medical diagnoses [22]. Applications of AI that have been trained using a machine learning model from a large variety of case reports for various diseases have the potential to accurately find patterns from unseen data inputs to diagnose a disease. Such AI models can improve diagnostic accuracy, reduce the rate of misdiagnosis, and ensure that patients receive appropriate treatment in a timely manner. Consequently, minimizing diagnostic inaccuracy contributes to standardizing the diagnosis process, leading to consistency in diagnoses across different healthcare providers in various environments.

The benefits of specialized AI models for diagnosing diseases are plentiful and multi-faceted. The ultimate beneficiary, patients, can receive more accurate diagnoses, leading to better management, culminating in more efficient treatment of their condition. For example, using AI for imaging diagnosis against COVID-19 significantly reduced the diagnosis time for AI-assisted doctors compared to routine diagnosis [23]. Thus, health care providers can leverage AI tools to support their diagnostic processes, upholding their commitment to delivering high-quality patient care. This is especially beneficial in areas with limited access to health care professionals, where widespread access to these AI medical diagnostic tools can reduce the burden on the health care system in the region. The financial and operational costs associated with misdiagnosis and inappropriate treatment can be reduced with accurate and consistent specialized AI models.

In accordance with our findings, a recent study using ChatGPT-3.5 found a 67% success rate in initial diagnosis and a 59% success rate in medication recommendation [24]. However, the same study noted a high rate of unnecessary or harmful medication recommendations that occurred in 85% of the trials and 59% in trials after a correct diagnosis compared to primary care providers where the corresponding rates ranged from 28 to 64% [24], raising concerns about patient safety. Therefore, our findings are not in isolation, and reflect general concerns regarding the exclusive use of LLMs for disease diagnosis.

A retrospective study analyzing the diagnostic accuracy of ChatGPT-4V for acute stroke using diffusion MRI images found that ChatGPT-4 with vision (ChatGPT-4V) had an accuracy of 88.3% [25]. When comparing this to text-only models, the addition of visual data interpretation significantly enhanced diagnostic accuracy, demonstrating the potential for multimodal AI models to provide more comprehensive and accurate assessments rather than text-based models alone.

While AI has the potential to revolutionize the diagnostic process by offering significant benefits to the broad medical community, the shortcomings of AI models in diagnosing diseases cannot be ignored. Training and effective functioning of AI models require a large amount of data. Racially-, ethnically- and gender-biased data would compromise the diagnostic accuracy of the model. According to Yale’s School of Medicine, “Health care algorithms that power AI may include bias against underrepresented communities and thus amplify existing racial inequality in medicine” [26]. Accordingly, datasets and case reports must be applicable to individuals of various racial, ethnical, and gender backgrounds to perform accurate diagnoses across diverse populations. Furthermore, ethical and legal concerns surrounding patient privacy and data security will likely exist regarding the use of patient information to train the AI model. The accountability for diagnostic errors made by AI and the transparency-confidentiality balance of the AI decision-making process highlight the ambiguity shrouding this technology. Therefore, there is a need for robust regulations to ensure the responsible integration of AI models into diagnostic practice.

The findings of this study may have the potential to be applied internationally; however, the relevance may vary based on the resources available in different health care settings. In low-resource settings, LLMs such as ChatGPT-3.5 could serve to improve diagnostic capabilities, but their effectiveness may vary depending on the availability and quality of imaging and other diagnostic techniques. Conversely, in high-resource health care settings, these tools could enhance diagnostic efficiency, provided they are effectively integrated into clinical workflows.

The integration of AI into diagnostics raises legal and ethical concerns. As the medical community becomes increasingly reliant on AI tools, issues surrounding liability and responsibility come to light, particularly when misdiagnosis occurs. The “black box” nature of AI algorithms complicates causality assessment in malpractice cases, necessitating frameworks to address liability matters and ensure accountability [27]. Furthermore, given the sensitive nature of medical information used to train AI models, data privacy and security are essential. It is crucial that AI systems adhere to data protection regulations to maintain patient trust and protect their information. Ultimately, while AI shows promise in improving diagnostic efficiency and accuracy, its implementation must be approached with caution.

### 4.1 Strengths

The systematic data collection procedure involved using the reputable PubMed database for full-text case reports published in the last decade and varied in complexity with reports from diverse geographical regions ensure that the case information presented to ChatGPT-3.5 reflected recent clinical practices for diagnostic criteria. Disabling the memory function contributed to reducing the bias presented to the AI model from previous interactions, forcing the model to rely solely on the clinical information provided, with the exclusion of the case diagnosis.

Though the 78.6% accuracy rate achieved in this study is promising, it also highlights the limitations associated with using AI for medical diagnosis. The remaining 21 cases where GPT-3.5 failed to accurately diagnose MS indicate that there are still significant areas for improvement. These errors could stem from several factors, including variability in presentation of clinical information and laboratory data in different case reports, the inherent complexity of diagnosing MS and potential gaps in the training datasets used to develop the AI model. Future studies should focus on understanding these discrepancies and refining the AI algorithms to further increase diagnostic accuracy.

### 4.2 Limitations

The current study is limited to textual data, primarily consisting of patient medical histories, clinical and laboratory findings, and symptom descriptions. As a result, ChatGPT-3.5 cannot leverage the full spectrum of available diagnostic information even if they are present in the case report, inevitably leading to less accurate diagnoses. In fact, in over 90% of people with MS, diagnosis is confirmed using MRI scans, which reveal the exact location and size of inflammation, damage, and scarring [28]. Since ChatGPT-3.5 does not currently possess the capability to analyze images, MRI scans of brain lesions cannot be included to support the AI model’s analysis and diagnosis. ChatGPT-4o and subsequent versions are capable of analyzing text, images, and audio, which likely enhance the diagnostic accuracy [27]. ChatGPT is one of the many increasingly popular LLMs, alongside Microsoft’s Copilot and Google’s Gemini. Each of these LLMs has been trained on different datasets and the findings of this study based on the abilities of ChatGPT-3.5 are not representative of different LLMs currently in the market. As of September 13, 2024, ChatGPT-3.5 has been shut down. The rapidly changing AI landscape highlights the limitations of using an outdated model for a diagnostic study. Since the release of ChatGPT-4o was announced subsequent to designing this study and data collection, it was deemed a deviation from the original study design to adopt ChatGPT-4o. Our future studies will aim to test new AI platforms to determine whether they offer better accuracy for diagnostic medicine.

## 5 Conclusion

This study illustrates how AI models such as ChatGPT-3.5 can be utilized to diagnose MS through analysis of text-based case reports. With an accuracy of 78.6%, the findings suggest that AI is capable of interpreting clinical and laboratory data for medical diagnosis. However, the 21.4% error margin highlights the limitations of ChatGPT-3.5 and areas needing improvement in AI diagnostic tools, which may stem from variations in the clinical data presentation, gaps in training data, the limitation of ChatGPT-3.5 to use only text-based datasets, and the complexity of MS diagnosis. The medical community must prioritize research efforts, validation processes, and careful consideration of the legal and ethical aspects related to utilizing AI in health care. By taking this approach, health care professionals can harness the capabilities of AI to improve patient care while reducing the risks involved.
